# Differential genetic background control state-dependent courtship ultrasonic vocalizations in mice

**DOI:** 10.1007/s10048-025-00857-0

**Published:** 2025-11-01

**Authors:** Saeyeon Na, Jia Ryoo, Chang Bum Ko, Daesoo Kim

**Affiliations:** Department of Brain and Cognitive Sciences, Advanced Institute of Science and Technology (KAIST), Daejeon, 34141 Republic of Korea

**Keywords:** Ultrasonic vocalization, Courtship behavior, Appetitive state, Consummatory state, Genetic control

## Abstract

In male mice, courtship behaviors encompass distinct appetitive and consummatory phases, accompanied by ultrasonic vocalizations (USV) that vary with courtship progression and exhibit strain-specific patterns. Despite these differences, how genetics contribute to state- and strain-dependent USV variations remain unclear. We examined USV syllable patterns during courtship in inbred C57BL/6J (B6) and 129S4/SvJae (129) mouse strains, along with their mixed-background F2 offspring. During appetitive behaviors, such as body and anogenital sniffing, B6 and 129 males produced distinct USV. F2 males emitted USV reflecting combinations of B6 and 129 patterns, correlative with the continuous similarity to each strain. In contrast, all groups emitted strikingly similar USV during mounting. These findings suggest that appetitive and consummatory phases are governed by distinct genetic mechanisms, shaped by evolutionary pressures for vocalization diversity during appetitive behaviors and conservation during consummatory behaviors, supporting adaptation to selection pressures that favor flexibility in mate attraction while ensuring consistency in mating success.

**Graphical abstract:**

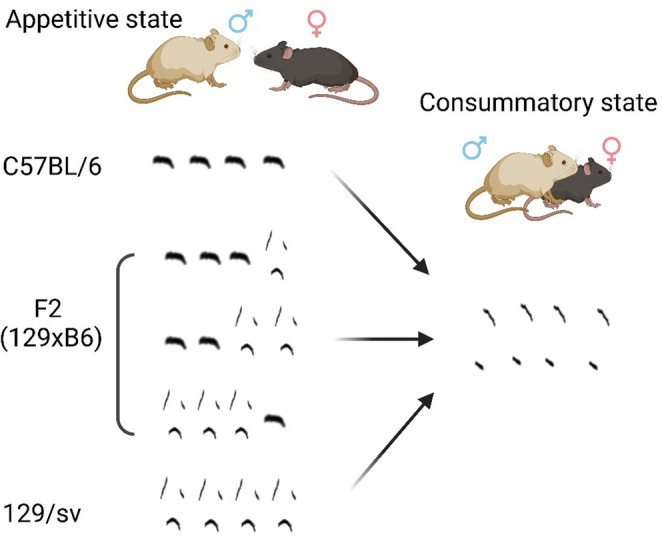

**Supplementary Information:**

The online version contains supplementary material available at 10.1007/s10048-025-00857-0.

## Introduction

Of the complex nature of social interactions, exhibiting appropriate behaviors at the right time and place with the right intensity is crucial. The courtship behavior of male mice starts from the appetitive state, during which body and anogenital investigation is used to recognize and select an appropriate mate. This is followed by the consummatory state, which starts with a series of mount attempts [1–14]. In this paper, we call these specific behavioral states simply *actions* in sexual behavior.

Ultrasonic vocalization (USV) is the major means of rodent vocal communication. USV pattern differs by social context. In particular, mating is one of the most extensively studied condition where USV is emitted [13–17]. More recently, the unit of interest has changed from quantitative values to syllable type usage to capture the pattern of USV [15], leading to the development of tools for automatic detection of the syllable types [18]. The general classification of USV consists of 12 categories: chevron, reverse chevron, down-frequency modulation, up-frequency modulation, flat, short, complex, one step up, one step down, two-step, multi-step, and noisy or harmonic syllable [19, 20].

The actions of sexual behavior could be explained by the specific syllable type used. In several strains including C57BL/6J (B6), male mice majorly emitted simple (chevron, reverse chevron, down-frequency modulation, up-frequency modulation, flat, short, complex) syllables during appetitive state, whereas during consummatory state, stepped (one step up, one step down, two-step, multi-step, and noisy or harmonic) syllables were dominantly produced [13, 14]. Despite this discovery of major syllable types used in each action, the factors that make this alteration remain elusive.

Genetic background is another major factor influencing USV syntax, along with social and internal cues [17, 19, 21, 22]. In particular, a study revealed that USV syntax is under genetic control by showing that B6 and 129S4/SvJae (129) majorly produced different types of syllables during sexual behavior and that their second filial generation (F2) showed all 129-like, B6-like, mixed-type, or novel-type phenotype [22]. However, during which action among sexual behavior genetic background affects USV syntax has been poorly understood.

Thus, we investigated the genetic effect on distinct actions using USV and cross- bred mice. The probability of occurrence (PO) of each syllable was measured during the total time, body sniffing, anogenital sniffing, and mounting after identifying each syllable using VocalMat software [18]. The effects of the genetic back- ground and action on syllable usage (PO) were analyzed using two-way mixed ANOVA and a linear regression. Furthermore, we also identified syllables whose usage significantly changed due to these effects by investigating the genetic influence on distinct actions.

## Results

### USV syllable usage during courtship actions is genetically influenced

**Fig. 1:**
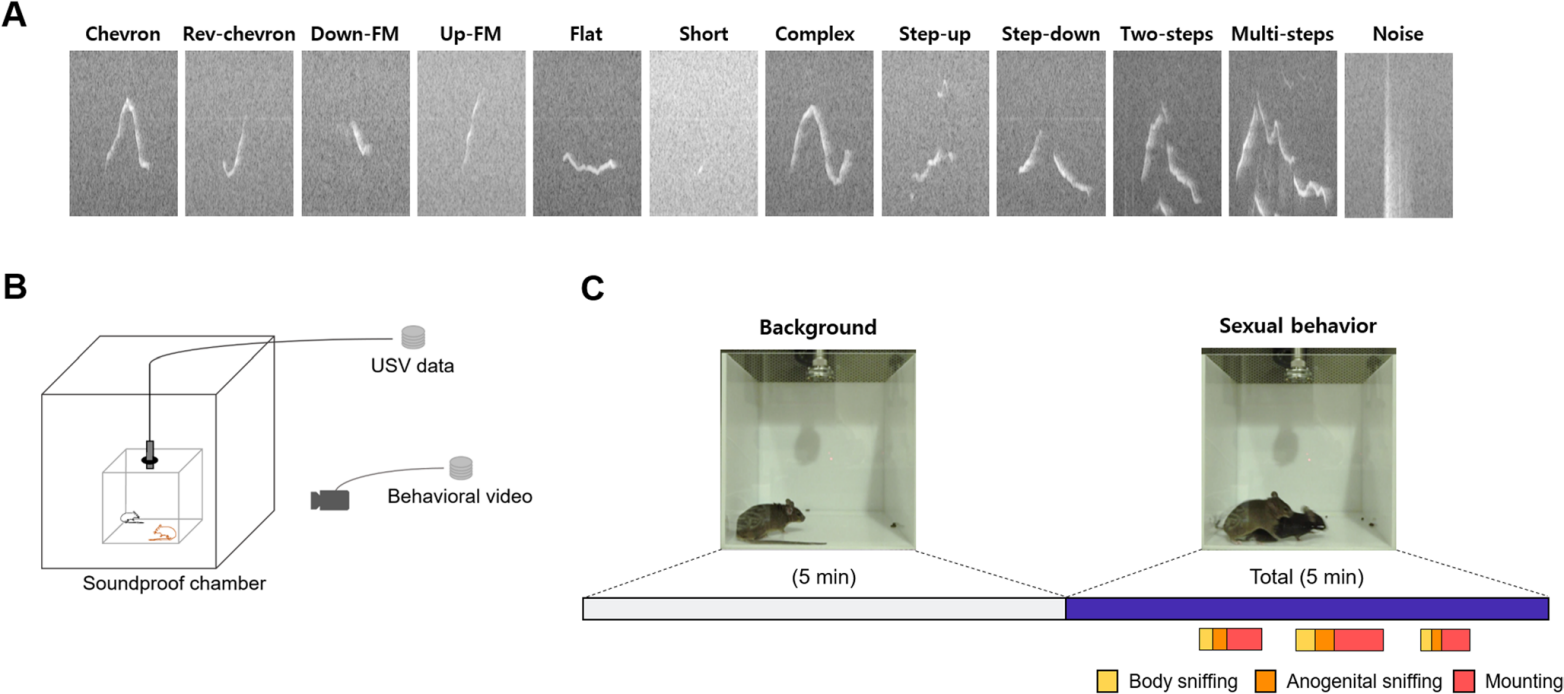
Experimental scheme. (**a**) Sample syllable classification of chevron, reverse chevron, down-frequency modulation, up-frequency modulation, flat, short, complex, step-up, step-down, two-step, multi-step, and noise from VocalMat results. (**b**) Experimental design. (**c**) Representative sample video frames from background and sexual behavior recording. The colors indicate the actions for which the POs of each syllable type were calculated and were as follows: violet, total duration; yellow, body sniffing; orange, anogenital sniffing; red, mounting. Images generated using Microsoft power point

Since male mice are known to produce specific ultrasonic vocalizations (USV) during appetitive and consummatory actions, we aimed to identify the effect of genetics on these state-dependent USV. We used the VocalMat software [18] to identify action-dependent USV syllables of C57BL/6J (B6) mice, 129S4/SvJae (129) mice, and their F2 progeny in the presence of freely moving female intruders (Fig. 1). We isolated 12 classical USV syllable types as previously described [19, 20, 22] and excluded noise from further analysis (Fig. 1a).

Since the genotype of F2 mice depends on contributions from 129 and B6 strains, direct comparison of USV syllable usage between F2 and parental strains is statistically inappropriate [23]. Instead, we devised a genetic similarity score that reflected the relative genetic proximity of a given F2 individual to the 129 or B6 strains. The genetic similarity score was calculated using chevron and up-frequency modulation (up-FM) syllables, known to be dominant USV syllable markers for B6 and 129 mice, respectively [21, 22]. The genetic similarity score was designed to be higher when the USV syllable-related genetics were closer to 129 and lower when they were closer to B6.

To examine how syllable usage varied among animals of different genetic backgrounds, we examined the probabilities of occurrence (POs) for nine syllable types (from among the 12 syllables, chevron, up-FM, and noise were excluded) during distinct actions of sexual behavior (Fig. 1b and c).

Comparison of syllable usage between B6 and 129 mice during the total duration revealed statistically significant differences (*p <* 0.05) across various syllable x strain pairs (*p <* 0.00002, two-way mixed ANOVA, Table S1−5), underscoring the presence of strain-specific vocalization patterns.

**Fig. 2:**
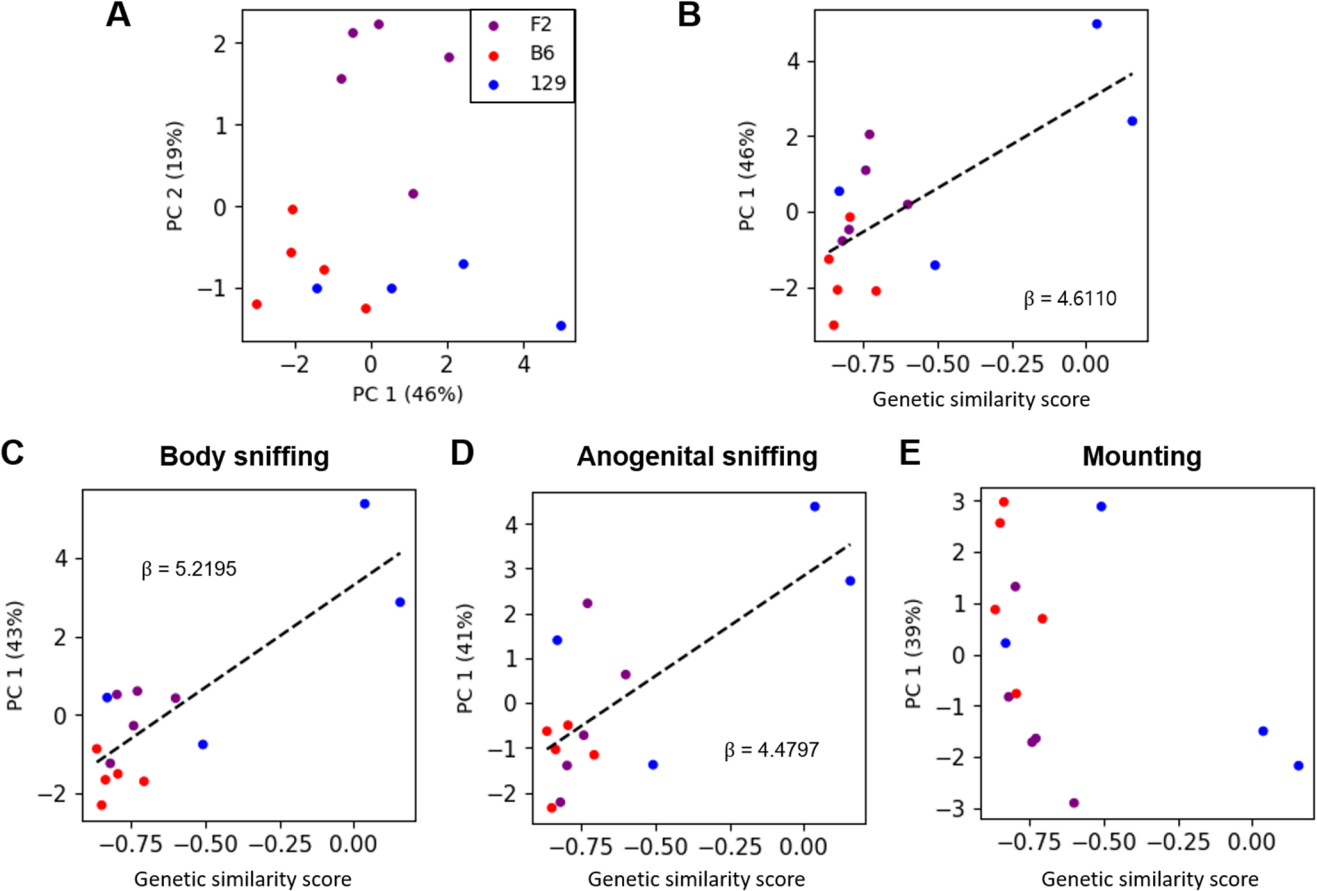
Genetic similarity scores exhibit linear relationships with dimensionally reduced syllable POs for total duration, body sniffing, and anogenital sniffing. (**a**) Scatter plot of the first and second principal components of the POs for syllables. PC1 explained about 46% of the variance and PC2 explained about 19%. Scatter plot of the genetic similarity score and PC1 values for (**b**) total duration, (**c**) body sniffing, (**d**) anogenital sniffing, and (**e**) mounting. Each point represents an individual animal, with genotypes indicated by the color: purple, F2 (*n* = 5); red, B6 (*n* = 5); and blue (*n* = 4). A linear regression was employed; Bonferroni correction was used for the analyses presented in c, d, and e. Dashed line indicates *p <* 0.05. Images generated using python notebook

To test whether syllable usage over the total duration of courtship behaviors varied according to genetic similarity in the offspring generation, we included F2 mice and assessed whether there was a continuous linear relationship between genetic similarity and syllable usage. The differences in genetic similarity comes from the random assortment of related loci. We employed a linear regression with the genetic similarity score of each mouse and the first principal component (PC1) from principal component analysis (PCA) results obtained from the nine assessed syllables (Fig. 2a). This regression identified a statistically significant linear relationship (*p <* 0.05) between the genetic similarity score and the dimensionality-reduced overall syllable usage (*p* = 0.0005, Fig. 2b, Table S6). B6, 129, and F2 genetic similarity scores and the summarized syllable usage exhibited a linear relationship during the total sexual behavior period. The investigation of F2 mice was done to provide evidence of inheritance, which is an essential feature of a genetic factor, and to observe USV syllable patterns in heterozygous mice.

To assess whether strain-specific syllable usage was associated with specific mating-related actions, POs were calculated during three actions to capture the difference in appetitive (body sniffing and anogenital sniffing) versus consummatory (mounting) actions. To assess for syllable usage differences between B6 and 129 strains, we compared the POs of these strains for each action. We found that B6 mice and 129 mice showed statistically significant differences (*p <* 0.05, Bonferroni correction) in the interaction of strain and syllable usage during body sniffing and anogenital sniffing, but not during mounting (two-way mixed ANOVA, body sniffing: *p <* 0.00005; anogenital sniffing: *p <* 0.000006; mounting: *p >* 0.4; Table S1). Regression showed that there was a significant linear relationship (*p <* 0.05, Bonferroni correction) between the genetic similarity score and PC1 during body sniffing and anogenital sniffing, but not during mounting (body sniffing: *p <* 0.0000006, anogenital sniffing: *p <* 0.0006, mounting: *p >* 0.3, Fig. 2c-e, Table S7). Thus, we observed a strain difference and linear relationship in the appetitive stage of sexual behavior but not the consummatory stage.

## Two-step and flat syllable usage diversity during sniffing

**Fig. 3:**
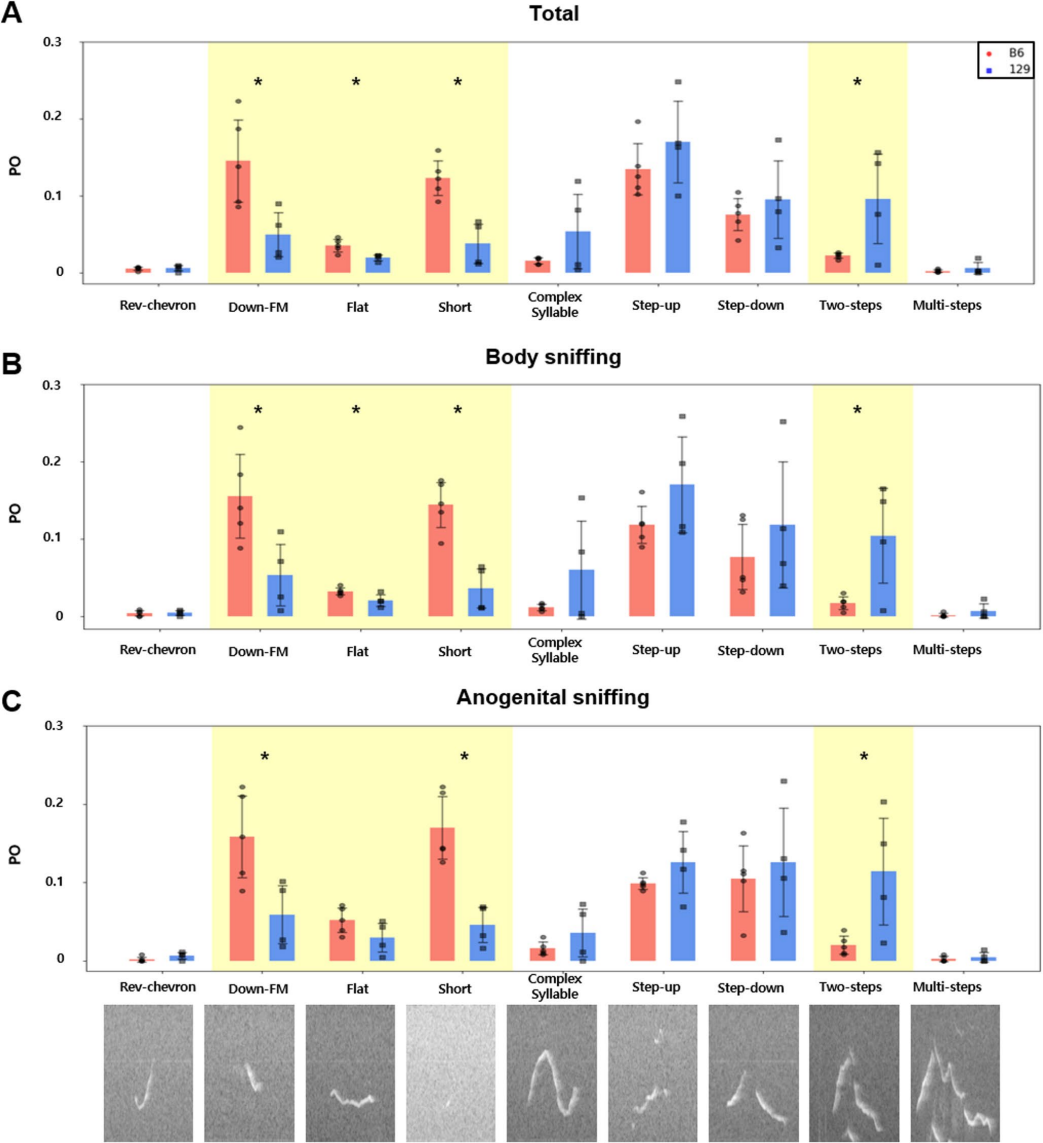
POs differed significantly between B6 and 129 strains for down-FM, flat, short, and two-step during total duration and different actions. Between-strain differences in POs were compared during (**a**) total duration (**b**) body sniffing (**c**) anogenital sniffing. Each black point represents the PO of that syllable in each male mouse (B6, circle, *n* = 5; 129, square, *n* = 4). Bar represents the mean POs of B6 and 129 mice for each syllable. Yellow shading represents the syllables with POs that differed significantly between B6 and 129 mice during at least6one action. All data presented as mean $\:\pm\:$SD. *p < 0.05. Images generated using Microsoft power point and python notebook

To identify which syllable(s) largely contributed to the pattern of strain-specific syllable usage in the actions prior to mounting, we conducted post-hoc tests. Tukey’s HSD (honestly significant difference) test was applied between strains for the PO of each syllable during each action. The POs of down-FM and short syllables were higher in B6 mice during the total, body sniffing, and anogenital sniffing actions. Two-step syllable usage was higher in 129 mice during total, body sniffing, and anogenital sniffing while flat syllable usage was higher in B6 mice during total duration and body sniffing. The POs of the remaining syllables did not show any statistically significant difference between B6 and 129 mice (Tukey’s HSD test, Fig. 3a-c, Table S8). During the appetitive stage of sexual behavior, B6 mice used more down-FM and short syllables, while 129 mice used more two-step syllables. During body sniffing, B6 mice predominantly used the flat syllable. Flat and two-step syllables demonstrated strain-dependent usage during the appetitive phase.

**Fig. 4:**
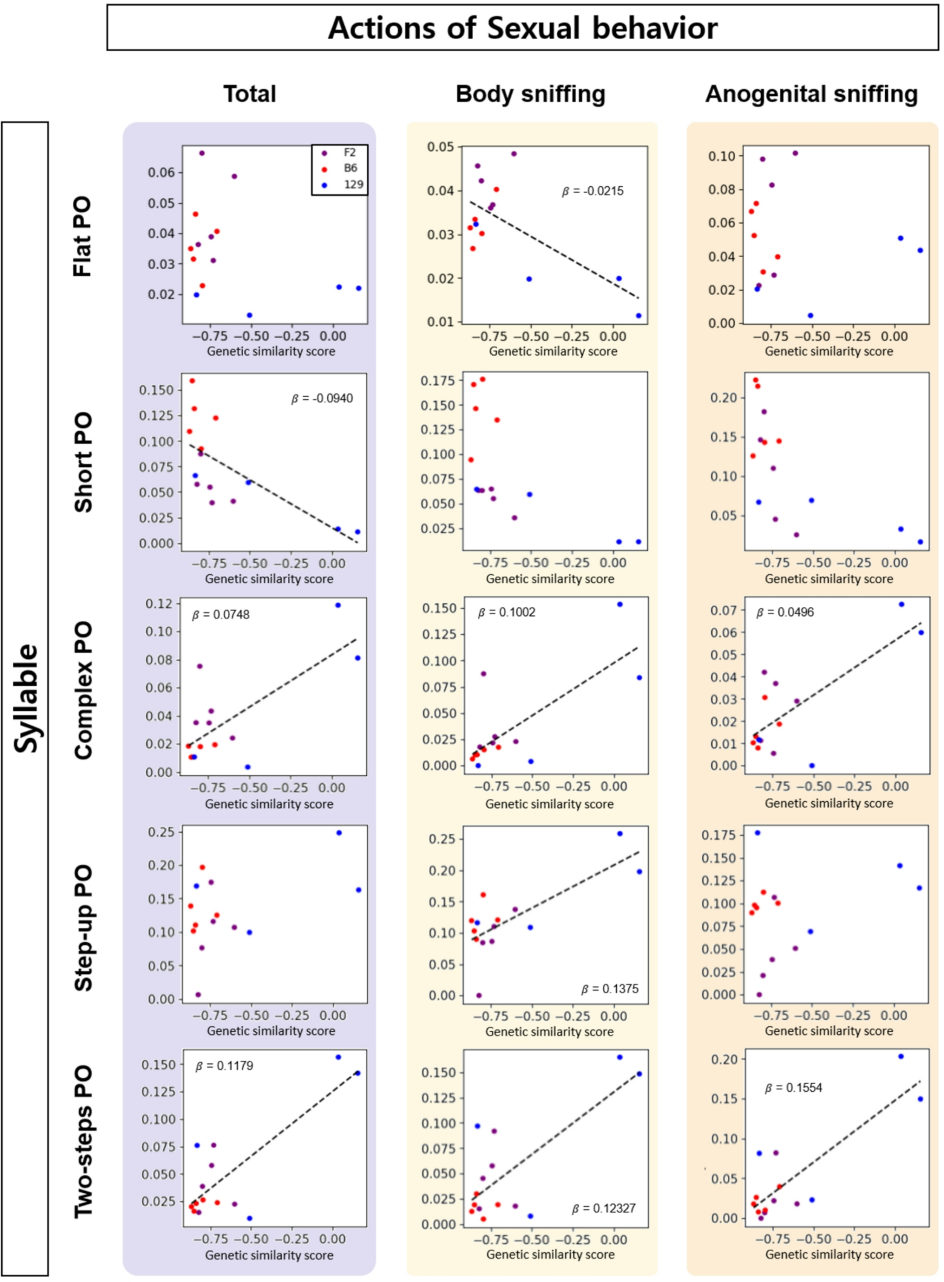
POs of flat, short, complex, step-up, and two-step syllables correlate with genetic similarity score. During total duration (light violet), the POs of short, complex, and two-step syllables showed linear relationships with the genetic similarity score; during body sniffing (light yellow), those of the flat, complex, step-up, and two-step syllables showed this relationship; and during anogenital sniffing (light orange), those of the short and two-step syllables showed this relationship. Each point represents an animal; purple, F2 (*n* = 5); red, B6 (*n* = 5); and blue, 129 (*n* = 4). A linear regression was applied, and Bonferroni correction was used. Dashed line indicates *p <* 0.05. Images generated using Microsoft power point and python notebook

A linear regression was fit between the genetic similarity score and the PO of each syllable. The goal was to identify syllables that contributed to the linear relationships of the genetic similarity score and syllable usage during total duration, body sniffing and anogenital sniffing phases (Fig. 4). Bonferroni correction was applied to determine the significance (*p <* 0.05) of linearity. Several syllables’ PO showed continuous change due to the genetic similarity score within F2. The POs of complex and two-step syllables showed positive linear relationships with genetic similarity scores across all three actions, suggesting that they represented 129-like syllables. The step-up syllable showed a similar pattern, but only during body sniffing. Male F2 mice that were genetically closer to the 129 used more complex and two-steps syllables during mating, particularly during body and anogenital sniffing, and used more step-up syllables during body sniffing. Short and flat syllables exhibited negative linear relationships with the genetic similarity score, suggesting that they represented B6- like syllables. Male F2 mice that were genetically closer to the B6 strain used more of the short syllable during the total duration and more of the flat syllable during body sniffing, while those closer to the 129 used fewer of these syllables during these actions. (Fig. 4, Table S7). B6-like F2 male mice used more of the flat syllable and 129-like male mice used more of the two-step syllable.

## Discussion

### Genetic basis for USV syllable usage diverging during the appetitive stage and remaining homogeneous during the consummatory stage

Our study shows how genetic background influences ultrasonic vocalization (USV) syllable usage during various courtship actions in inbred male mice. Studies have shown that genetic background and behavioral state [19] are the primary factors of syllable syntax. However, the interplay between these factors has not yet been studied in depth. We found that, in sexual behavior of male mice, USV syllable usage differed by the genetic background during appetitive actions but not consummatory actions. B6 mice, 129 mice, and their F2 descendants produced similar syllable patterns during mounting but not during appetitive actions.

Specifically, flat and two-step syllables demonstrated strain-dependent usage during the appetitive phase. B6-like F2 male mice used more of the flat syllable and 129-like male mice used more of the two-step syllable. These syllables were distinct from those exhibiting strain-specificity throughout the entire course of sexual behavior, as well as from those primarily used during the appetitive phase. The usage of the flat syllable demonstrated genetic dependency during body sniffing, but was not significantly related to the genetic similarity score during the entirety of the mating process. In contrast, the short syllable displayed B6-like usage consistently throughout the total duration, but not during body sniffing. This pattern corroborates previous reports of B6-like usage of the short syllable and 129-like usage of the Chevron syllable during the total duration [22]. Furthermore, two-step syllable usage was influenced by genetic background during the appetitive state, but was not predominant. Instead, the simpler step-up, step-down, down-FM, and short syllables predominated. This aligns with earlier reports indicating that simple and short syllables (chevron, reverse chevron, down-frequency modulation, up-frequency modulation, flat, short) predominate during appetitive states [13, 14, 16, 24].

Historically, it has been suggested that innate behaviors consist of two different phases: appetitive and consummatory behaviors, by ethologists [16, 25–28]. The diverse appetitive and fixed consummatory behaviors in sexual and feeding behavior has been observed in various species, such as rodents, Japanese quail, pigeons, fish, and invertebrates [25–31]. However, there were difficulties in investigating the genetic control of two different behavioral states in those non-model animals. Thus, we used two inbred strains of mouse and their F2 progeny to figure out the genetic basis of two states. C57BL/6J (B6) and 129S4/SvJae (129) strain share same ancestry, being derived from the same subspecies *M. m. domesticus*. However, a phylogenetic analysis has shown that they have significant genetic distance [32]. Our results suggest that during the selection his- tory of these two strains, selection and evolutionary divergence [33] may have occurred around appetitive behaviors, whereas there was relative conservation of mounting motor pathways, possibly due to differential selection pressures such as female choice [34, 35].

Our findings highlight the intricate relationship between genetic background and actions in inbred male mouse courtship behavior related to USV syllable usage, emphasizing that genetic background is related to diversity in appetitive USV but homogeneity in consummatory USV. These findings suggest that appetitive and consummatory courtship phases are governed by distinct genetic mechanisms, shaped by evolutionary pressures for vocalization diversity during exploratory behaviors and conservation during consummatory behaviors, supporting adaptation to selective pressures that favor flexibility in mate attraction while ensuring consistency in mating success. Male selection by females may evolve toward preferring various male partners while transferring genes through mating depends on consistent male ability.

## Materials and methods

### Experimental model details

#### Animals

All procedures were conducted according to the Korean Advanced Institute of Science and Technology (KAIST) Guidelines for the Care and Use of Laboratory Animals and were approved by the Institutional Animal Care and Use Committee. Mice (Mus musculus) were maintained under a 12-h light/dark cycle at 23^*◦*^C with ad libitum access to food and water. Male C57BL/6J mice (B6, RRID: IMSR JAX:000664; *n* = 29), 129S4/SvJae mice (129, RRID: MGI:2164439; *n* = 25), and second filial generation mice (F2, *n* = 38) of the two strains were bred at KAIST (Daejeon, Korea). Mice were habituated on the hands of experimenters for 10 min each day for 5 days. The mice were then isolated in transparent housing cages for at least 7 days before courtship USV recording, and exposed to a double soundproof recording chamber (20 × 20 × 20) for 30 min for 3 days to minimize anxiety. Recording was done on 10- to 18-week- old male mice. For each recording, an estrous B6 wildtype female at least 8 weeks of age was introduced to the test chamber. Estrous stage was confirmed by visual inspection of the vagina [36]. B6 females were used in all of the sessions because the syllable compositions of B6 and 129 males was previously shown to be independent of the female strain [22].

## Method details

### Courtship USV induction

Female introduction was used to induce courtship USV, since this strategy induces the production of USV by males more effectively than other stimulants [14]. On the recording day, a male mouse was placed in one side of a transparent recording chamber for 30 min without recording for habituation. Then, 5 min of background recording was obtained and a female was introduced to the test chamber. The male mouse was allowed to investigate the female for 5 min (Fig. 1c). Emitted USV were recorded in the double-soundproof chamber (Fig. 1b). Only data from males that exhibited proper mounting behavior and had audio recordings free of any disturbing noise was used to analyze USV (*n* = number of animals; F2, *n* = 5; B6, *n* = 5; 129, *n* = 4). The experimenter was aware of the group allocation (strain) during the experimental process, outcome assessment, and data analysis.

### USV and courtship behavior data acquisition

USV were recorded using a 1/4-inch microphone and amplified using a preamplifier and main amplifier (Bruel and Kjaer Inc., Denmark). Behavior was simultaneously recorded with a video camera.

### Quantification and statistical analysis

#### Analysis of USV structure

Each male selected for analysis had more 100 USV syllable events (the mouse that had the least syllable events had 799 events and was F2). USV syllables were labeled using VocalMat. Produced USV were analyzed across four time periods: the total duration, body sniffing, anogenital sniffing, and the mounting action (Fig. 1b). To determine the timepoints for onset and offset of each action session, time-series video data were manually scored to onset and duration of three types of behavior: body sniffing, anogenital sniffing, and mounting. Syllables that belonged to the specific action time periods were labeled using the onset and duration data with Python. The video and audio data were synchronized using clap sound. All USV syllables that were detected by VocalMat was assumed to be coming from male mice. Although during male-female sexual behavior, female mice predominantly emit squeaks, which is not detected as a syllable in this paper’s criteria, this excludes the chance that some of the syllables were produced by female mice.

Probability of occurrence (PO) was adopted from general syntax analysis in Chabout et al. [15] and calculated as:

$$\begin{array}{c}\:Probability\:\left(occurrence\:of\:a\:syllable\:type\right)=\:\\\frac{total\:number\:of\:occurrences\:of\:a\:syllable\:type}{total\:number\:of\:syllables\:of\:all\:types}\end{array}$$

### Generation of genetic similarity score

$$\:\overrightarrow{{\mathbf F2}_{\mathbf n}}\:=\:x^\ast\overrightarrow{{\mathbf B6}_{\mathbf r\mathbf e\mathbf g\mathbf r\mathbf e\mathbf s\mathbf s\mathbf e\mathbf d}}+\:y\boldsymbol\ast\overrightarrow{{129}_{\mathbf r\mathbf e\mathbf g\mathbf r\mathbf e\mathbf s\mathbf s\mathbf e\mathbf d}}$$

$$\:\mathbf A=\left[\overrightarrow{{\mathbf B6}_{\mathbf r\mathbf e\mathbf g\mathbf r\mathbf e\mathbf s\mathbf s\mathbf e\mathbf d}},\:\overrightarrow{{129}_{\mathbf r\mathbf e\mathbf g\mathbf r\mathbf e\mathbf s\mathbf s\mathbf e\mathbf d}}\right]^T,\:\mathbf B=\:\overrightarrow{{\mathbf F2}_{\mathbf n}},\:\mathbf X=\left[x,y\right],\:\mathbf A\mathbf X=\mathbf B$$

$$\:GS\:(=genetic\:score)=\frac xy$$

The genetic similarity scores of F2 individuals with respect to the B6 and 129 strains were produced using Up-FM and Chevron PO, which were reported to be majorly used by B6 and 129 mice, respectively [21, 22]. The Up-FM PO x Chevron PO data points for B6 and 129 mice were fitted into a linear regression. The F2 data point vector was represented with the B6 and 129 regression vectors.

The genetic similarity score was set to be higher when the genetic background was closer to 129 mice and lower when the genetic background was closer to B6 mice.

### Statistical analysis

The significance level was defined as *p<*0.05. ANOVA with two-way mixed factors was used to analyze the total duration and time period-dependent differences in vocalization data, as applied using Python 3.9.17 pingouin (0.5.3) mixed anova. Strain was used as between-factor and syllable identity was used as within-factors (repeated measure). Tukey’s HSD test was used for pairwise multiple comparison, as applied with statsmodels (0.14.0). Sphericity test, normality test (Shapiro-Wilk), and equal variance test were done on the dependent variable (PO). All data presented in Figure 3 are given as mean SD.

For principal component analysis (PCA), feature matrices were created wherein each row showed a given individual mouse at each time period; each column represented one of the 9 syllables (excluding Chevron and Up-FM which were used for the genetic similarity score, and also excluding Noise), and each entry reflecting the PO of the given syllable type at the indicated time period for that individual. Data were standardized by preprocessing with StandardScaler of Scikit-learn. PCA was done using Python Scikit-learn (1.4.0) decomposition PCA for two principal components (PCs). The PC1-PC2 scatter plot was plotted in Python with color used to indicate the genetic background identity. The explained variance of each PC were calculated using pca.explained_variance_ratio. The genetic similarity score and PC1 or PO data for each syllable were fitted into the linear regression (gaussian family generalized linear model with identity link function); constants were added to genetic similarity score before fitting.

identity link function: g($\:\mu\:$) = $\:\alpha\:+\:\beta\:\mathbf x$

Python statsmodels (0.14.0) GLM was used to perform the fitting. Normality was tested for dependent variables (PC1 or PO for each syllable).

### Key resources table

**Table:** 

REAGENT or RESOURCE	SOURCE	IDENTIFIER
Experimental model: Organism/strain		
Mouse: C57BL/6J (B6, RRID: IMSR_JAX:000664; *n* = 31),	The Jackson Laboratory	RRID: IMSR_JAX:000664
Mouse: 129S4/SvJae (129, RRID: MGI:2164439; *n* = 25)	The Jackson Laboratory	RRID: MGI:2,164,439
Software and algorithm		
Python 3.9.17	Python Software Foundation	https://www.python.org/

## Electronic supplementary material

Below is the link to the electronic supplementary material.

Supplementary Material 1

## Data Availability

This study did not generate new unique reagents. All data and code reported in this paper will be shared by the lead contact upon request. Any additional information required to reanalyze the data reported in this paper is available from the lead contact upon request. Further information and requests for resources should be directed to and will be fulfilled by the lead contact, Daesoo Kim (daesoo@kaist.ac.kr).
