# Supplementary material for: Differential genetic background control state-dependent courtship ultrasonic vocalizations in mice
Source: Neurogenetics. 2025 Nov 1;26(1):77. doi: 10.1007/s10048-025-00857-0 (PMC12578711; doi:10.1007/s10048-025-00857-0)
Supplement: Supplementary file 1 — Supplementary Material 1 [file 10048_2025_857_MOESM1_ESM.docx]

Differential genetic background control state-dependent courtship ultrasonic vocalizations in mice

Saeyeon Na^1^, Jia Ryoo^1^, Chang Bum Ko^1^, Daesoo Kim^1*^

1*Department of Brain and Cognitive Sciences, Advanced Institute of Science and Technology (KAIST), 34141, Daejeon, Korea, Republic of Korea.

*Corresponding author(s). E-mail(s): [daesoo@kaist.ac.kr](mailto:daesoo@kaist.ac.kr)

Table S1. Statistics of two-way mixed ANOVA of syllables PO during total duration, body sniffing, anogential sniffing, and mounting (Strain x Syllable). *p<0.05.

| Compared group | Test used | Source | P value | DF1 | DF2 | F | Period |
| --- | --- | --- | --- | --- | --- | --- | --- |
| B6 vs 129 | Two-way mixed ANOVA | Strain | 0.7664 | 1 | 7 | 0.0954 | Total duration |
|  |  | Syllable | <0.0000000000003 | 8 | 56 | 18.9619 |  |
|  |  | Interaction | <0.00002* | 8 | 56 | 6.1755 |  |
|  | Two-way mixed ANOVA  Bonferroni correction | Strain | 0.8975 | 1 | 7 | 0.017835 | Body sniffing |
|  |  | Syllable | <0.0000000003 | 8 | 56 | 13.034854 |  |
|  |  | Interaction | <0.00005* | 8 | 56 | 5.953680 |  |
|  |  | Strain | 0.3954844 | 1 | 7 | 0.819246 | Anogenital sniffing |
|  |  | Syllable | <0.000000000009 | 8 | 56 | 15.583585 |  |
|  |  | Interaction | <0.000006* | 8 | 56 | 7.139715 |  |
|  |  | Strain | 0.8106103 | 1 | 7 | 0.061933 | mounting |
|  |  | Syllable | <0.000000002 | 8 | 56 | 11.684135 |  |
|  |  | Interaction | 0.407557123 | 8 | 56 | 1.634259 |  |

Table S2. Total number of produced syllables of individual B6, 129, and F2 mice with genetic identity average.

| Genetic identity | Total number of produced syllables | Genetic identity average |
| --- | --- | --- |
| B6 | 834 | 1317.4 |
|  | 1088 |  |
|  | 1686 |  |
|  | 1428 |  |
|  | 1551 |  |
| 129 | 1185 | 1203 |
|  | 1316 |  |
|  | 1164 |  |
|  | 1147 |  |
| F2 | 799 | 1003 |
|  | 905 |  |
|  | 1063 |  |
|  | 1244 |  |
|  | 1004 |  |

Table S3. Statistics of basic quantitative features of ultrasonic vocalization syllables of F2 mice.

|  | Duration (ms) | Mean intensity total (dB) | Max freq main (kHz) | Mean freq main (kHz) | Bandwidth (Hz) |
| --- | --- | --- | --- | --- | --- |
| chevron | 64.10$\pm$0.992 | -87.03$\pm$0.2215 | 87.228$\pm$0.444 | 74.124$\pm$0.38 | 29097$\pm$415.5 |
| Rev-chevron | 49.55$\pm$3.35 | -88.32$\pm$0.6941 | 84.111$\pm$1.996 | 72.241$\pm$1.393 | 21751$\pm$1860 |
| Down-FM | 40.73$\pm$3.58 | -91.03$\pm$0.4847 | 79.11$\pm$1.304 | 70.487$\pm$1.059 | 15319$\pm$1251 |
| Up-FM | 29.89$\pm$0.42 | -91.81$\pm$0.1687 | 84.992$\pm$0.388 | 75.281$\pm$0.313 | 20721$\pm$370.1 |
| flat | 27.40$\pm$1.71 | -91.36$\pm$0.4603 | 68.378$\pm$0.908 | 64.738$\pm$0.782 | 7165$\pm$746.2 |
| short | 8.36$\pm$0.42 | -96.45$\pm$0.3369 | 72.590$\pm$0.899 | 71.026$\pm$0.858 | 3353$\pm$375.97 |
| complex | 95.62$\pm$3 | -88.76$\pm$0.4208 | 89.301$\pm$0.898 | 74.363$\pm$0.622 | 22731$\pm$945.6 |
| Step-up | 99.25$\pm$3.24 | -87.13$\pm$0.252 | 87.481$\pm$0.721 | 66.417$\pm$0.439 | 36731$\pm$713.6 |
| Step-down | 60.23$\pm$1.9 | -88.65$\pm$0.3814 | 91.555$\pm$0.861 | 72.462$\pm$0.578 | 33996$\pm$870.4 |
| Two-steps | 89.07$\pm$4.45 | -88.65$\pm$0.3814 | 94.851$\pm$1.118 | 72.883$\pm$0.842 | 40272$\pm$1227 |
| Multi-steps | 127.83$\pm$10.96 | -88.06$\pm$0.9374 | 94.255$\pm$3.048 | 67.372$\pm$2.224 | 45849$\pm2627$ |
| noise | 37.88$\pm$3.35 | -99.67$\pm$0.2416 | 64.979$\pm$0.617 | 57.629$\pm$0.404 | 13338$\pm$597.2 |

Table S4. Statistics of basic quantitative features of ultrasonic vocalization syllables of B6 mice.

|  | Duration (ms) | Mean intensity total (dB) | Max freq main (kHz) | Mean freq main (kHz) | Bandwidth (Hz) |
| --- | --- | --- | --- | --- | --- |
| chevron | 37.75$\pm$1.14 | -86.25$\pm$0.2727 | 95.484$\pm$0.825 | 83.306$\pm$0.595 | 25071$\pm$907.7 |
| Rev-chevron | 47.81$\pm$4.86 | -82.39$\pm$0.9878 | 88.433$\pm$1.953 | 77.694$\pm$1.372 | 19100$\pm$1968 |
| Down-FM | 27.51$\pm$1.2 | -86.71$\pm$0.2523 | 91.411$\pm$0.725 | 80.730$\pm$0.496 | 19190$\pm$720.9 |
| Up-FM | 21.98$\pm$0.37 | -86.11$\pm$0.1518 | 90.898$\pm$0.388 | 80.782$\pm$0.268 | 18544$\pm.407.4$ |
| flat | 24.12$\pm$1.19 | -82.96$\pm$0.4265 | 77.642$\pm$0.695 | 74.992$\pm$0.613 | 5619$\pm$664.5 |
| short | 7.95$\pm$0.28 | -89.62$\pm$0.1764 | 84.655$\pm$0.666 | 82.210$\pm$0.616 | 4875$\pm$379.2 |
| complex | 78.54$\pm$6.17 | -82.49$\pm$0.6256 | 90.714$\pm$1.710 | 76.956$\pm$1.198 | 26035$\pm$1894 |
| Step-up | 97.23$\pm$2.76 | -85.00$\pm$0.1670 | 95.356$\pm$0.614 | 72.922$\pm$0.394 | 38265$\pm$639.8 |
| Step-down | 39.91$\pm$1.77 | -87.10$\pm$0.2118 | 101.027$\pm$0.717 | 82.457$\pm$0.515 | 33778$\pm$862.6 |
| Two-steps | 75.61$\pm$3.70 | -85.74$\pm$0.3154 | 104.400$\pm$1.168 | 79.027$\pm$0.757 | 44041$\pm$1203 |
| Multi-steps | 164.35$\pm$28.64 | -83.57$\pm$0.7921 | 102.140$\pm$4.408 | 73.654$\pm$1.743 | 48295$\pm$4155 |
| noise | 33.19$\pm$1.66 | -93.93$\pm$0.1234 | 75.222$\pm$0.531 | 66.398$\pm$0.453 | 16701$\pm$432.5 |

Table S5. Statistics of basic quantitative features of ultrasonic vocalization syllables of 129 mice.

|  | Duration (ms) | Mean intensity total (dB) | Max freq main (kHz) | Mean freq main (kHz) | Bandwidth (Hz) |
| --- | --- | --- | --- | --- | --- |
| chevron | 58.90$\pm$1.18 | -84.72$\pm$0.2082 | 91.966$\pm$0.491 | 76.042$\pm$0.299 | 30251$\pm$664.3 |
| Rev-chevron | 53.19$\pm$7.58 | -86.36$\pm$1.3262 | 78.518$\pm$3.929 | 68.116$\pm$2.631 | 17931$\pm$2941 |
| Down-FM | 34.29$\pm$2.96 | -88.5329$\pm$0.4060 | 84.813$\pm$1.247 | 72.964$\pm$0.830 | 20674$\pm$1614 |
| Up-FM | 27.57$\pm$0.98 | -87.74$\pm$0.3219 | 82.087$\pm$0.744 | *74.031*$\pm$*0.588* | 16107$\pm$730.2 |
| flat | 24.22$\pm$2.61 | -86.00$\pm$0.7685 | *71.775*$\pm$*1.444* | 68.184$\pm$1.181 | 6510$\pm$1094 |
| short | 7.84$\pm$0.59 | -90.78$\pm$0.4146 | 74.461$\pm$1.264 | 72.655$\pm$1.263 | 3687$\pm$613.1 |
| complex | *104.71*$\pm2.86$ | -82.12$\pm$0.4002 | 96.898$\pm$1.279 | 74.734$\pm$0.588 | 38466$\pm$1521 |
| Step-up | 85.62$\pm$1.72 | -87.08$\pm$0.2277 | 95.719$\pm$0.706 | 69.348$\pm$0.363 | 43482$\pm$729.2 |
| Step-down | 60.38$\pm$2.91 | -87.22$\pm$0.3392 | 99.720$\pm$1.118 | 76.365$\pm$0.779 | 39305$\pm$1280 |
| Two-steps | 73.28$\pm$1.54 | -85.89$\pm$0.2906 | 106.062$\pm$0.715 | 76.451$\pm$0.619 | 50978$\pm$906.3 |
| Multi-steps | 104.70$\pm$8.95 | -83.09$\pm$2.1748 | 104.156$\pm$7.729 | 72.623$\pm$5.957 | *49133*$\pm$*7591* |
| noise | 41.54$\pm$1.32 | -93.85$\pm$0.1130 | 80.785$\pm$0.525 | 66.935$\pm$0.347 | 23802$\pm$589.6 |

Table S6. Genetic similarity scores of individual B6, 129, and F2 mice.

| Genetic identity | Genetic similarity score |
| --- | --- |
| B6 | -0.7942629586325809 |
|  | -0.8652247216559844 |
|  | -0.8361472253871381 |
|  | -0.8488831373225344 |
|  | -0.7061688720318842 |
| 129 | 0.15622780760195934 |
|  | -0.8302497464839984 |
|  | 0.0370505705579021 |
|  | -0.5077758429505189 |
| F2 | -0.8198625925559977 |
|  | -0.7973058473697786 |
|  | -0.7284739884285524 |
|  | -0.5998035256164301 |
|  | -0.7411816429504425 |

Table S7. Statistics of generalized linear model (GLM; Gaussian family, identity link function) fitting of PC1 of 9 syllables’ PO and syllables (Reverse-chevron; Down-FM; Flat; Short; Complex; Step-up, Step-down, Two-steps, Multi-steps) PO during total duration, body sniffing, anogenital sniffing, and mounting (Strain x Syllable). *p<0.05. Fig: Figure, Indep. Var.: independent variable, Dep. Var.: dependent variable, R^2^: Pseudo R-square (CS), Bonf: Bonferroni correction, GS: genetic score.

| Fig | Indep. Var. (PO) | Indep. Var.: period | Dep. Var. | Test used | P value ($\boldsymbol{\alpha}$) | P value ($\boldsymbol{\beta}$) | $\boldsymbol{\alpha}$ | $\boldsymbol{\beta}$ | N | R^2^ |
| --- | --- | --- | --- | --- | --- | --- | --- | --- | --- | --- |
| 2B | PC1 of 9 syllables’ | Total duration | GS | GLM | 0.0017 | 0.0005* | 2.9254 | 4.6110 | 14 | 0.5896 |
| 2C | PC1 of 9 syllables’ | Body sniffing | GS | GLM, Bonf | <0.000009 | <0.0000006* | 3.3114 | 5.2195 | 14 | 0.8592 |
| 2D | PC1 of 9 syllables’ | Anogenital sniffing | GS | GLM, Bonf | 0.0025 | 0.0006* | 2.8421 | 4.4797 | 14 | 0.6329 |
| 2E | PC1 of 9 syllables’ | Mounting | GS | GLM, Bonf | 0.4335 | 0.3102 | -1.6083 | -2.5350 | 14 | 0.1819 |
| - | Rev-chev | Total duration | GS | GLM, Bonf | 0.5652 | 1 | 0.007427 | -0.001627 | 14 | 0.01710 |
| - | Down-FM | Total duration | GS | GLM, Bonf | 1 | 0.9567 | 0.023204 | -0.084173 | 14 | 0.1793 |
| 4 | Flat | Total duration | GS | GLM, Bonf | 0.0561 | 1 | 0.023989 | -0.016722 | 14 | 0.1319 |
| 4 | Short | Total duration | GS | GLM, Bonf | 1 | 0.0079 | 0.015029 | -0.093976 | 14 | 0.5517 |
| 4 | Complex | Total duration | GS | GLM, Bonf | <0.00000005 | 0.0019 | 0.083358 | 0.074756 | 14 | 0.6299 |
| 4 | Step-up | Total duration | GS | GLM, Bonf | <0.00000006 | 0.5074 | 0.185463 | 0.086180 | 14 | 0.2377 |
| - | Step-down | Total duration | GS | GLM, Bonf | 0.0402 | 1 | 0.084796 | -0.001551 | 14 | 0.01133 |
| 4 | Two-steps | Total duration | GS | GLM, Bonf | <0.000000000003 | <0.00001 | 0.125039 | 0.117875 | 14 | 0.8200 |
| - | Multi-steps | Total duration | GS | GLM, Bonf | 0.0078 | 0.4501 | 0.008660 | 0.007198 | 14 | 0.2485 |
| - | Rev-chev | Body sniffing | GS | GLM, Bonf | 1 | 1 | 0.007697 | 0.000702 | 14 | 0.01204 |
| - | Down-FM | Body sniffing | GS | GLM, Bonf | 1 | 1 | 0.016806 | -0.099322 | 14 | 0.2042 |
| 4 | Flat | Body sniffing | GS | GLM, Bonf | 0.0027 | 0.0421 | 0.01876 | -0.02154 | 14 | 0.5162 |
| 4 | Short | Body sniffing | GS | GLM, Bonf | 1 | 0.1108 | 0.014752 | -0.106222 | 14 | 0.4510 |
| 4 | Complex | Body sniffing | GS | GLM, Bonf | <0.000002 | 0.0024 | 0.097691 | 0.100225 | 14 | 0.6704 |
| 4 | Step-up | Body sniffing | GS | GLM, Bonf | <0.0000000000000002 | 0.0015 | 0.208182 | 0.137533 | 14 | 0.6913 |
| - | Step-down | Body sniffing | GS | GLM, Bonf | 0.8957 | 1 | 0.084997 | -0.003098 | 14 | 0.01144 |
| 4 | Two-steps | Body sniffing | GS | GLM, Bonf | <0.00000005 | 0.0015 | 0.130363 | 0.123172 | 14 | 0.6894 |
| - | Multi-steps | Body sniffing | GS | GLM, Bonf | 0.4071 | 1 | 0.008676 | 0.007379 | 14 | 0.1515 |
| - | Rev-chev | Anogenital sniffing | GS | GLM, Bonf | 1 | 1 | 0.008513 | 0.002072 | 14 | 0.01820 |
| - | Down-FM | Anogenital sniffing | GS | GLM, Bonf | 1 | 1 | 0.027781 | 0.082423 | 14 | 0.1318 |
| 4 | Flat | Anogenital sniffing | GS | GLM, Bonf | 0.3722 | 1 | 0.045496 | -0.008531 | 14 | 0.01875 |
| 4 | Short | Anogenital sniffing | GS | GLM, Bonf | 1 | 0.0579 | 0.021029 | 0.140536 | 14 | 0.4955 |
| 4 | Complex | Anogenital sniffing | GS | GLM, Bonf | <0.000000005 | 0.0019 | 0.056422 | 0.049645 | 14 | 0.6795 |
| 4 | Step-up | Anogenital sniffing | GS | GLM, Bonf | 0.0016 | 1 | 0.114174 | 0.042965 | 14 | 0.08890 |
| - | Step-down | Anogenital sniffing | GS | GLM, Bonf | 0.3279 | 1 | 0.109010 | 0.009029 | 14 | 0.01276 |
| 4 | Two-steps | Anogenital sniffing | GS | GLM, Bonf | <0.00000000002 | <0.000002 | 0.147723 | 0.155416 | 14 | 0.8762 |
| - | Multi-steps | Anogenital sniffing | GS | GLM, Bonf | 0.0060 | 0.2416 | 0.007946 | 0.007940 | 14 | 0.3931 |

Table S8. Statistics of post-hoc Tukey’s HSD test of syllables (Reverse-chevron; Down-FM; Flat; Short; Complex; Step-up, Step-down, Two-steps, Multi-steps) PO during total duration, body sniffing, anogenital sniffing, and mounting (Strain x Syllable).

| Figure | Comparison groups | period | syllable | Mean differences | 95% Confidence intervals | Significance level | P-values | Reject (<0.05) |
| --- | --- | --- | --- | --- | --- | --- | --- | --- |
| 3A | B6 vs 129 | Total duration | Rev-chevron | -0.0006 | (-0.0057, 0.0045) | 0.05 | 0.7858 | False |
| 3A | B6 vs 129 | Total duration | Down-FM | 0.0956 | (0.0166, 0.1747) | 0.05 | 0.0243 | True |
| 3A | B6 vs 129 | Total duration | Flat | 0.0159 | (0.0043, 0.0275) | 0.05 | 0.0141 | True |
| 3A | B6 vs 129 | Total duration | Short | 0.0854 | (0.0428, 0.1281 | 0.05 | 0.0021 | True |
| 3A | B6 vs 129 | Total duration | Complex | -0.038 | (-0.0962, 0.0201) | 0.05 | 0.1657 | False |
| 3A | B6 vs 129 | Total duration | Step-up | -0.0353 | (-0.1131, 0.0424) | 0.05 | 0.318 | False |
| 3A | B6 vs 129 | Total duration | Step-down | -0.0198 | (-0.0864, 0.0469) | 0.05 | 0.5056 | False |
| 3A | B6 vs 129 | Total duration | Two-steps | -0.074 | (-0.1441, -0.0039) | 0.05 | 0.0412 | True |
| 3A | B6 vs 129 | Total duration | Multi-steps | -0.0037 | (-0.0131, 0.0057) | 0.05 | 0.3839 | False |
| 3B | B6 vs 129 | Body sniffing | Rev-chevron | -0.0008 | (-0.0068, 0.0053) | 0.05 | 0.7761 | False |
| 3B | B6 vs 129 | Body sniffing | Down-FM | 0.1019 | (0.015, 0.1888) | 0.05 | 0.0276 | True |
| 3B | B6 vs 129 | Body sniffing | Flat | 0.0116 | (0.0008, 0.0223) | 0.05 | 0.0386 | True |
| 3B | B6 vs 129 | Body sniffing | Short | 0.1076 | (0.0581, 0.1572) | 0.05 | 0.0013 | True |
| 3B | B6 vs 129 | Body sniffing | Complex | -0.0484 | (-0.1245, 0.0277) | 0.05 | 0.1764 | False |
| 3B | B6 vs 129 | Body sniffing | Step-up | -0.0515 | (-0.1322, 0.0292) | 0.05 | 0.175 | False |
| 3B | B6 vs 129 | Body sniffing | Step-down | -0.0412 | (-0.154, 0.0715) | 0.05 | 0.4157 | False |
| 3B | B6 vs 129 | Body sniffing | Two-steps | -0.0873 | (-0.1614, -0.0132) | 0.05 | 0.0271 | True |
| 3B | B6 vs 129 | Body sniffing | Multi-steps | -0.0057 | (-0.0173, 0.0059) | 0.05 | 0.2847 | False |
| 3C | B6 vs 129 | Anogenital sniffing | Rev-chevron | -0.0047 | (-0.0112, 0.0018) | 0.05 | 0.1327 | False |
| 3C | B6 vs 129 | Anogenital sniffing | Down-FM | 0.0993 | (0.0167, 0.182) | 0.05 | 0.025 | True |
| 3C | B6 vs 129 | Anogenital sniffing | Flat | 0.0223 | (-0.008, 0.0526) | 0.05 | 0.1247 | False |
| 3C | B6 vs 129 | Anogenital sniffing | Short | 0.1237 | (0.0635, 0.1839) | 0.05 | 0.0018 | True |
| 3C | B6 vs 129 | Anogenital sniffing | Complex | -0.0199 | (-0.0583, 0.0186) | 0.05 | 0.2614 | False |
| 3C | B6 vs 129 | Anogenital sniffing | Step-up | -0.0271 | (-0.0753, 0.0212) | 0.05 | 0.2262 | False |
| 3C | B6 vs 129 | Anogenital sniffing | Step-down | -0.0207 | (-0.1208, 0.0794) | 0.05 | 0.6393 | False |
| 3C | B6 vs 129 | Anogenital sniffing | Two-steps | -0.0938 | (-0.1768, -0.0108) | 0.05 | 0.0319 | True |
| 3C | B6 vs 129 | Anogenital sniffing | Multi-steps | -0.0024 | (-0.0108, 0.006) | 0.05 | 0.5254 | False |
